# Improving source estimation of retinotopic MEG responses by combining data from multiple subjects

**DOI:** 10.1162/imag_a_00265

**Published:** 2024-08-12

**Authors:** Paavo Hietala, Ilmari Kurki, Aapo Hyvärinen, Lauri Parkkonen, Linda Henriksson

**Affiliations:** Department of Neuroscience and Biomedical Engineering, Aalto University School of Science, Espoo, Finland; Department of Psychology and Logopedics, Faculty of Medicine, University of Helsinki, Helsinki, Finland; Department of Computer Science, Faculty of Science, University of Helsinki, Helsinki, Finland; MEG Core and AMI Centre, Aalto NeuroImaging, Aalto University School of Science, Espoo, Finland

**Keywords:** magnetoencephalography, joint analysis, inverse problem, eLORETA, minimum Wasserstein estimates, retinotopic mapping

## Abstract

Magnetoencephalography (MEG) is a functional brain imaging modality, which measures the weak magnetic field arising from neuronal activity. The source amplitudes and locations are estimated from the sensor data by solving an ill-posed inverse problem. Commonly used solutions for these problems operate on data from individual subjects. Combining the measurements of multiple subjects has been suggested to increase the spatial resolution of MEG by leveraging the intersubject differences for increased information. In this article, we compare 3 multisubject analysis methods on a retinotopic mapping dataset recorded from 20 subjects. The compared methods are eLORETA with source-space averaging, minimum Wasserstein estimates (MWE), and MWE with source-space averaging. The results were quantified by the geodesic distances between early (60–100 ms) MEG peak activations and fMRI-based retinotopic target points in the primary visual cortex (V1). By increasing the subject count from 1 to 10, the median distances decreased by 6.6–9.4 mm (33–46%) compared with the single-subject median distances of around 20 mm. The observed peak activation locations with multisubject analysis also comply better with the established retinotopic maps of the primary visual cortex. Our results suggest that higher spatial accuracy can be achieved by pooling data from multiple subjects. The strength of MWE lies in individualized and sparse source estimates, but in our data, averaging eLORETA estimates across individuals in source space outperformed MWE in spatial accuracy.

## 1 Introduction

Magnetoencephalography (MEG) is a functional brain imaging technique, which measures the magnetic field arising from the electrical activity in the brain. Inferring the underlying source activity from the MEG measurements is centered on solving an ill-posed inverse problem. As an infinite amount of source configurations can produce the same MEG signal, imposing constraints based on prior information or assumptions is required to solve the problem. Common priors include MRI-based source spaces, assuming a level of synchronicity between neighboring sources and selecting the solution with minimum energy (Becker et al., 2015).

The inverse problem is commonly solved for each subject individually. In the distributed source imaging approach, a large number of source dipoles are placed in fixed locations on the cortex and their amplitudes are then estimated by minimizing a cost function. These methods include, for example, minimum norm estimates (MNE;Hämäläinen & Ilmoniemi, 1984) and minimum current estimates (MCE;Matsuura & Okabe, 1995;Uutela et al., 1999) which use$\ell^{2}$and$\ell^{1}$regularization, respectively. These methods have been further developed to incorporate, for example, noise normalization and depth bias correction. Popular options include the LORETA family of methods (Pascual-Marqui et al., 1994;Pascual-Marqui, 2002,^2007^), dynamic statistical parametric mapping (dSPM;Dale et al., 2000), and mixed-norm estimates (MxNE;Strohmeier et al., 2014). The inverse problem can also be solved by employing a parametric approach, for example, by fitting individual dipoles on the cortex (Hämäläinen et al., 1993;Sarvas, 1987), or by utilizing beamforming or other source-scanning techniques (Mosher & Leahy, 1999;Van Veen et al., 1997).

The source estimates of individual subjects can be combined to compensate for the structural differences and to generalize the results to a larger population. Traditionally, the group-level inference has been conducted as a separate step after computing the individual source estimates. The subject-level data can be combined through either averaging in a common reference space or statistical inference. Statistical parametric and nonparametric mapping methods are commonly used for group analysis (Chau et al., 2004;Kilner & Friston, 2010;Nichols & Holmes, 2002). This strict division between the first-stage source estimation and second-stage group analysis is unlikely to leverage the full potential of a multisubject dataset. In theory, the structural and functional differences between the subjects introduce additional variation in the data which could be exploited in improving the source estimation accuracy of MEG. In fact, forming the group results by averaging individual subjects’ source estimates in a common reference frame has been shown to improve the spatial accuracy of MEG (Larson et al., 2014).

Joint analysis methods aim to improve the analysis outcomes by either creating a unified model for all subjects or by adjusting the individual models based on data from other subjects. For example, the variation between the subjects can be used to optimize the hyperpriors of a hierarchical Bayes model (Henson et al., 2011;Litvak & Friston, 2008). The inverse problems can be coupled even further, for example, by presenting them as a combined multitask learning problem, as recently shown byJanati, Bazeille, et al. (2020). If only sparsity is enforced for a multitask learner with, for example, an$\ell^{q}$norm, the model will assume a perfect overlap within active source dipoles of each subject (Janati et al., 2019). Minimum Wasserstein estimates (MWE;Janati, Bazeille, et al., 2020) relax this assumption through an optimal transport cost function. The method favors strong and focal source estimates and preserves the subjects’ individual signatures. Multisubject methods share an assumption of similar activation patterns between the subjects, hence the methods are not applicable to all datasets or experimental designs. Apart from source estimation, similar multisubject approaches have also been used to train MEG signal classifiers, which generalize well from the training group to a new individual (Csaky et al., 2023;Westner et al., 2018;Zubarev et al., 2019).

In this study, a retinotopic mapping dataset is analyzed with three different methods combining data from multiple subjects to quantify the benefits of multisubject approaches. The first applied method is the exact low-resolution brain electromagnetic tomography (eLORETA;Pascual-Marqui, 2007) with source-space averaging. The second method is minimum Wasserstein estimates and the third method is MWE with source-space averaging. These methods do not require predefined regions of interest (ROI) and they can be expected to localize a single focal source accurately. We compare the MEG source locations acquired with the different methods against functional magnetic resonance imaging (fMRI)-based target points, which have been determined from the subjects’ averaged retinotopic maps.

Retinotopic MEG responses provide a challenging, yet suitable test dataset for comparing the methods. As there are limited data on the real-world performance of the multisubject analysis methods, the comparison is restricted to the primary visual cortex (V1). The expected source locations are well defined, but the signal strength varies considerably by stimulus location. While the size of V1 shows great individual variability (Benson et al., 2022), the topology of retinotopic organization within V1 is consistent across subjects and follows the cortical folding (Hinds et al., 2008). Neuroimaging studies on retinotopy have mainly been done using fMRI and very accurate retinotopic maps have been extracted from the measurements (Larsson & Heeger, 2006;Sereno et al., 1995;Wandell et al., 2007). Here we compare the sources localized with MEG to fMRI-based target locations. MEG has also been used in retinotopic mapping although the cortical geometry and spatial resolution of the method increase the difficulty of obtaining accurate maps (Moradi et al., 2003;Nasiotis et al., 2017;Sharon et al., 2007).

## 2 Material and Methods

### 2.1 Dataset

The analyzed dataset had been collected byKurki et al. (2022)and it consisted of MEG, functional and structural MRI data collected from 20 volunteers (5 males, 15 females; mean age 22, range 19–29 years). The participants had no known neurological aberrations and normal or corrected-to-normal vision. Informed written consent had been given by all participants and the ethical approval for the original study had been given by the Aalto University Ethics Committee.

The multifocal retinotopic mapping stimulus used in the dataset is a modified version of the dartboard stimulus used byHenriksson et al. (2012)in an fMRI study. It consists of 24 regions arranged in 3 rings of 8 wedges. The radius of the inner circle measured 0.5 degrees, while the outer radii of the checkerboard rings were 2.3, 4.7, and 8.4 degrees, respectively. During the measurements, the participants had been instructed to press a button when the color of a fixation point in the middle of the screen changes. Each section flashed 258 times during each of the 7 runs, totaling 1806 flashes per stimulus region altogether. One run had been excluded for two subjects due to problems during the measurements. No other data segments were excluded. The stimulus is shown inFigure 1.

**Fig. 1.**
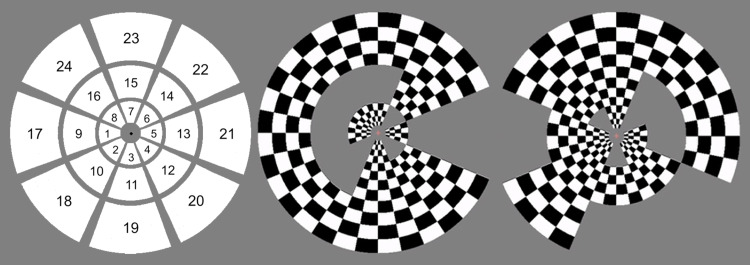
Visual description of the stimuli used in the analyzed dataset. Left: Stimulus region numbering. The stimulus consists of three rings of eight wedges to include both angle and eccentricity in the analysis. Middle and right: Two frames of the multifocal stimulus. The responses to the individual, temporally orthogonal stimulus blocks are separated using a general linear model.

The MEG data were acquired using a 306-channel system (Vectorview; MEGIN Oy, Espoo, Finland) in a 3-layer magnetically shielded room (Imedco AG, Hägendorf, Switzerland). The data were recorded at a sampling frequency of 1000 Hz and band-pass filtered between 0.1 and 330 Hz during the acquisition. A continuous head position indicator coil arrangement was used for head movement correction. Anatomical landmarks were collected with a Fastrak system (Polhemus, Inc., Colchester, VT, USA) for MEG–MRI coregistration. The stimuli were projected through a hole in the shielded room’s wall onto a back-projection screen in front of the subject with a 3-DLP projector (PT-D7700E; Panasonic Connect Co., Ltd., Tokyo, Japan). The stimulus presentation was controlled with PsychoPy 1.82.01 (Peirce, 2007).

The MRI data were collected with a 3 T whole-body MRI scanner (MAGNETOM Skyra; Siemens GmbH, Erlangen, Germany) and a 30-channel head coil. Two structural MRI images were taken per subject for a higher signal-to-noise ratio (SNR) and more optimal segmentation results. T1-weighted sequence was used for both images with a TR of 2530 ms, TE of 3.3 ms, and a slice count of 176. With field of view of 256 mm and acquisition matrix of 256×256 voxels, the voxel size was 1 mm^3^.

As a reference, fMRI data were collected with a similar 24-region multifocal stimulus for the same subjects who participated in the MEG experiment. Functional volumes were acquired using an echo planar imaging sequence with the following imaging parameters: TR of 2 s, 32 slices with 2.5-mm slice thickness (no gap), field of view 24 cm, imaging matrix 96 × 96, echo time 30 ms, and flip angle 70 degrees. The timing of the stimulus regions was as described byHenriksson et al. (2012). Four 4.5-minute multifocal fMRI runs were collected for each subject. One multifocal run consisted of 33 miniblocks each lasting 4 TRs.

### 2.2 Data preprocessing

Movement compensation and temporal signal space separation (tSSS;Taulu & Simola, 2006) were applied to the raw MEG data using MaxFilter 2.2 (MEGIN Oy, Espoo, Finland). Bad channels were identified manually and given as input to Maxfilter. Next, eye blink artifacts were removed with ICA (“runica” logistic infomax algorithm; one component removed based on correlation with EOG signal and visual inspection) and a lowpass filter (6th order butterworth) with a cutoff frequency of 45 Hz was applied in FieldTrip toolbox (Oostenveld et al., 2011). The MEG data were coregistered with the MRI images using MRIlab (MEGIN Oy, Espoo, Finland) with the help of the three anatomical landmarks (left and right preauricular points, and nasion). The alignment was fine-tuned based on the digitized locations of the coils and approximately 100 extra digitized points on the scalp surface. For details on the MEG data, please see the original article byKurki et al. (2022).

As multiple stimulus areas are visible at once in the multifocal retinotopic mapping paradigm, the sensor-level responses of each individual stimulus area were identified and separated using a general linear model (for details, seeKurki et al., 2022). The model’s finite impulse response basis functions covered the analyzed time window from -50 to 450 ms from stimulus onset. The modeling was done using custom MATLAB (Mathworks, Natick, MA, USA) code in the Neuroimaging methods group. The preprocessed evoked response matrix from MATLAB was converted into a set of MNE-Python’s evoked response objects and noise covariance matrices were computed for each subject from 1-minute resting-state recordings collected at the beginning of the measurement.

The scalp, skull, and brain meshes were segmented from the T1-weighted MRI images using FreeSurfer version 6.0.0 (Martinos Center for Biomedical Imaging, Charlestown, MA, USA). All further processing and analysis steps were performed using MNE-Python 0.22.0 (Gramfort et al., 2013) unless stated otherwise. The forward models were computed using a single-shell BEM model. To ensure similar dipole configuration between the subjects, the same template source space was used for all subjects. First, the template source space was created for the FreeSurfer’s*fsaverage*mesh using source spacing ico4, producing 2562 source points per hemisphere with a separation of 6.2 mm. The generated source space was then morphed to each subject’s individual anatomy by matching the sulci and gyri patterns of the inflated surfaces on an intermediate spherical surface (Fischl et al., 1999).

### 2.3 eLORETA and source-space averaging

In order to compare the joint analysis methods against a more traditional multisubject baseline, an analysis pipeline was implemented with individual subject inversion using eLORETA (Pascual-Marqui, 2007) and group averaging in source space. The improvement in source localization accuracy by averaging the results from a group of subjects was first quantified in an article byLarson et al. (2014). In the article the authors hypothesized that averaging would yield improved source localization results on group level through point-spread function overlap near the true activation sites. Although the original article utilized standardized low-resolution brain electromagnetic tomography (sLORETA;Pascual-Marqui, 2002) for the inverse solution, eLORETA was selected for its more focal source estimates, suppression of less significant sources, and comparable or better performance (Jatoi et al., 2014;Samuelsson et al., 2021). eLORETA is designed to have zero dipole localization error, hence it is likely to localize the small source areas accurately on V1.

The eLORETA source estimates are computed for each subject individually. The inverse operatorWis defined using an iteratively solved diagonal weighting matrix$D\in {\mathbb{R}}^{P\times P}$consisting of individual weights$d_{i}$for each source pointi, the leadfield matrix$L\in {\mathbb{R}}^{N\times P},$and the noise covariance matrix$\Sigma \in {\mathbb{R}}^{N\times N}$. HereNandPdenote the number of sensors and the number of source dipoles, respectively.

$$W=D^{-1}L^{T}{\left(LD^{-1}L^{T}\text{​}+\lambda^{2}\Sigma \right)}^{-1}$$ (1)

$$d_{i}=\frac{1}{x_{0}}\sqrt{L_{i}^{T}{\left(LD^{-1}L^{T}\text{​}+\lambda^{2}\Sigma \right)}^{-1}L_{i}}.$$ (2)

The addition of the term$\frac{1}{x_{0}}$with$x_{0}=1$Am was done to set the unit ofDto 1 / Am^2^as noted bySamuelsson et al. (2021). The inverse operator can then be used to fetch the source estimate$\hat{X}$from the sensor dataY:

$$\hat{X}=WY.$$ (3)

The regularization parameter$\lambda^{2}$was determined as$\lambda^{2}=\frac{1}{SNR^{2}}$using an estimated SNR of 2. The signal-to-noise ratio for each subject and stimulus was estimated and averaged over the duration of the visual response between 0 and 300 ms. The resulting SNR values were then averaged over all subjects and stimuli. The mean SNR over all subjects was 2.0 with a standard deviation of 1.3.

The individual source estimates for each subject were morphed back to the*fsaverage*mesh for further analysis. A pointwise arithmetic mean of the morphed source estimates was then calculated with 1, 5, 10, 15, and 20 subjects for each stimulus. To select the most representative subject as the “baseline” individual, the locations of peak activations were estimated using eLORETA. A geodesic distance was then computed between the peaks and fMRI-based targets for each subject and stimulus area. The subject with the median peak–target distance closest to the group median was selected as the representative individual. Distance mean, median, and standard deviation over all subjects and stimulus areas were 22.5 mm, 17.2 mm, and 20.2 mm, respectively. Subject 9’s median distance, 17.7 mm, was closest to the group result. The 5–20 subject groups were formed in a numerical order starting from the most representative subject to avoid biasing the results by favoring consistent data quality in the groups with a low number of subjects. Each subject group includes the individuals from the previous step; in other words, Subject 9 is included in the 5-subject results and subsequently all 5 subjects are included in the 10-subject results and so on.

### 2.4 Minimum Wasserstein estimates

The minimum Wasserstein estimates (Janati, Bazeille, et al., 2020) cast the inverse problem as a multitask regression problem, solving the source estimates for all subjects simultaneously. Even though the inverse solution is computed as one large operation, the source estimates for different subjects are not identical. Here these individual estimates are referred to as subject-specific MWE. The source estimates were computed for groups of 1, 5, 10, 15, and 20 subjects.

A basic multitask learner with sparsity constraints assumes an exact functional correspondence between the subjects, which is an untrustworthy prior (Janati et al., 2019). In MWE, this correspondence is relaxed by defining a regularization component which aims to minimize the Wasserstein distance between the activation barycenters. The Wasserstein distance is also known as earth mover’s distance, as it combines the distance and the difference in the measured quantity into a single transportation cost metric. In our case, the geodesic distances between active dipoles and their amplitudes are measured.

The minimum Wasserstein estimate is defined forSsubjects as

$${\hat{X}}^{1},...,{\hat{X}}^{S}=\underset{X^{1},...,X^{S}}{\text{argmin}}\frac{1}{2n}\sum_{s=1}^{S}{\left\|Y^{s}-L^{s}X^{s}\right\|}_{2}^{2}+\Omega \left(X^{1},...,X^{S}\right),$$ (4)

where superscriptsindicates a subject-specific variable. The regularization functionΩis defined with two components. The first component controls the sparsity of the source estimates with an$\ell^{q}$norm. Hereqwas set to 0.5. The second component controls the spatial variance between the subjects through averaged Wasserstein distancesWbetween the source estimates$X^{s}$and their barycenter$\bar{X}$. A generalized form of the Wasserstein distance is used, allowing for both positive and negative source amplitudes:

$$\Omega \left(X^{1},...,X^{S}\right)\overset{\text{def}}{=}\lambda {\left\|X^{s}\right\|}_{q}^{q}+\mu \underset{\bar{X}}{\text{min}}\frac{1}{S}\sum_{s=1}^{S}\;W\left(X^{s},\bar{X}\right).$$ (5)

While the regularization of eLORETA is based on the signal quality (SNR), the proper amount of regularization for MWE is found by adjusting the hyperparameters until the desired number of source dipoles is active. The main tuning hyperparameters of the minimum Wasserstein estimates areλandμ, which control the sparsity and spatial variance between the subjects, respectively. Optimizing the spatial variance hyperparameter is based on finding the value$\mu_{max}$, after which the source estimate is no longer sparse and spreads over the cortex. The source estimate is relatively stable for values under$\mu_{max}$as demonstrated inFigure 2. Here a modified binary search capable of finding the upper limit was used to find$\mu_{max}$. Following the suggestion of Janati, Bazeille, et al.,μwas set to$\frac{1}{2}\mu_{max}$as a safe heuristic.

**Fig. 2.**
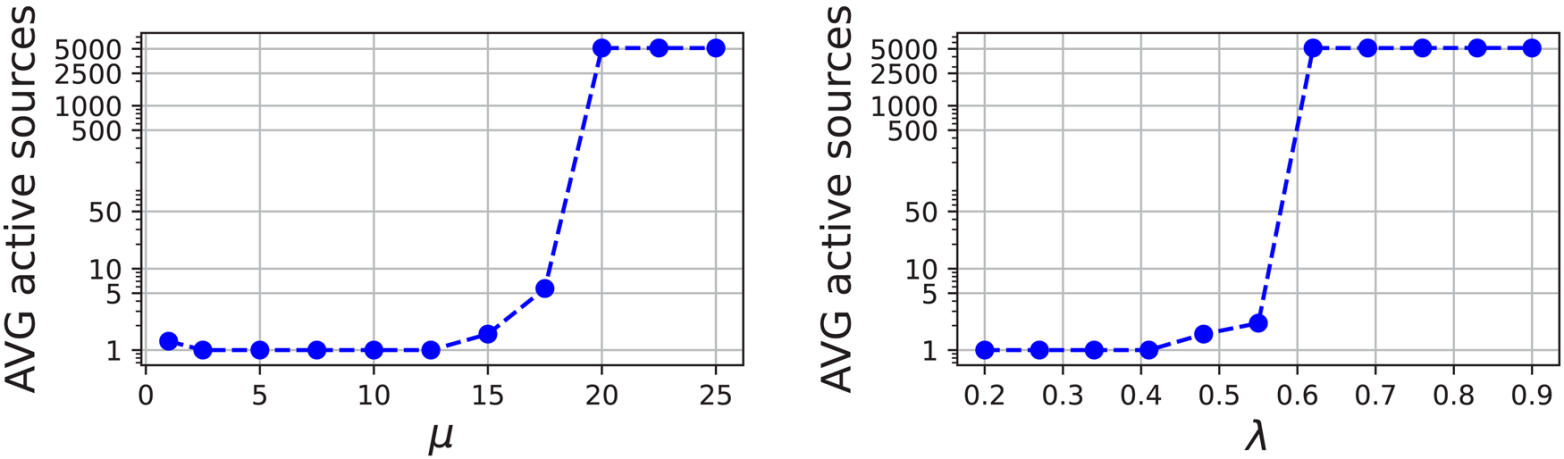
The relationships between MWE hyperparameters and average active source points for one stimulus. The sparsity limits are different for each stimulus and subject count. Left: Plot of the spatial variance hyperparameterμexhibits the transition value$\mu_{max}$between 15 and 20, after which the source estimate loses sparsity. Right: The sparsity hyperparameterλshows behavior similar toμwith a clear sparsity threshold at approximatelyλ=0.55.

The sparsity parameterλis determined as a fraction of the theoretical$\lambda_{max}=\frac{||L^{T}Y\text{​}|{|}_{\infty }}{n}$, after which the inverse solution should be uniformly 0 (Janati, Bazeille, et al., 2020). As withμ, a modified binary search is employed to find the value forλ∈[0,1]for which a suitable number of source points are active on average. The relationship betweenλand the number of active source points is shown in the right panel ofFigure 2. A target of three active points per subject was used for unilateral stimuli and six for stimuli on the vertical meridian, which were expected to cause bilateral activations. The numbers were selected based on localization accuracy and parameter search convergence. Optimal values for the main hyperparametersλandμvary per stimulus and have to be optimized individually for each case.

Additionally, the entropy regularization and marginal relaxation ofWcan be controlled with hyperparametersϵandγ. In practice, these hyperparameters have an effect on the smoothness of the results and the speed of model convergence (Janati, Bazeille, et al., 2020). They were given values of$\epsilon =\frac{5}{3P}$andγ=1based on the original publication.

The minimum Wasserstein estimates, along with a handful of other multitask regression methods, are implemented in the MuTaR package for Python (Janati, 2021). An MEG and EEG -friendly interface for generalized MuTaR solvers is provided by the GroupMNE package (Janati, Massich, & Gramfort, 2020). The GroupMNE package also includes preprocessing functions necessary to prepare the data for the source estimation. Version 0.0.1 of GroupMNE and version 0.0.1 of MuTaR were used.

### 2.5 MWE with source-space averaging

Averaging the subject-specific minimum Wasserstein estimates was selected as the third method in the comparison to test whether the results would be further improved from individual estimates. Similar to eLORETA, Euclidean averages were computed per vertex in source space. Unlike the implementation of eLORETA employed here, the minimum Wasserstein estimates also infer the direction of the dipole currents. To prevent opposite dipole moments from cancelling each other out, the averages were calculated on absolute values. The subjects were analyzed and averaged in the same numerical order as with the other two methods.

### 2.6 Extracting retinotopic targets from fMRI data

Functional MRI data were analyzed with SPM12 (Wellcome Department of Imaging and Neuroscience, London, UK) and custom MATLAB code. Functional volumes were corrected for interleaved acquisition order and for head motion. No spatial smoothing was applied. The data were denoised with the GLMdenoise toolbox Version 1.4 (Kay et al., 2013). The timing of the multifocal stimulus regions was entered as regressors of interest to the general linear model and convolved with the canonical hemodynamic response model. Six head motion parameters were included as additional regressors.

The multifocal responses were converted to eccentricity and polar angle maps. The data were averaged across individuals on the average cortical surface (*fsaverage*) using FreeSurfer and custom MATLAB code. Reference coordinates for the MEG results were extracted from the average retinotopic maps within V1. Both eccentricity and polar angle information was used to search for the closest location on the cortical surface. Polar angle information was weighted twice as much as eccentricity. The results are shown inFigure 3.

**Fig. 3.**
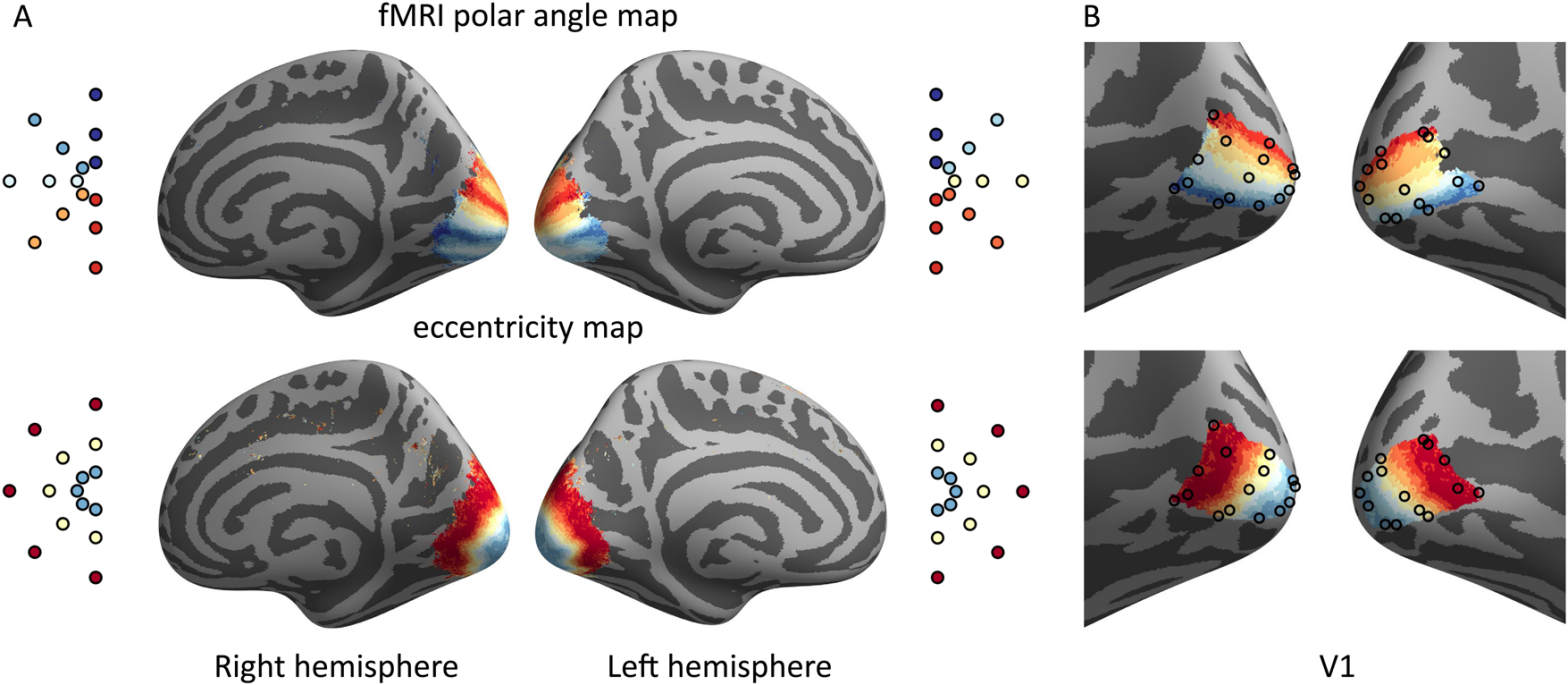
Retinotopic reference points from fMRI data. (A) Average polar angle and eccentricity maps for the 24-region multifocal stimulus. (B) Reference points within left and right V1 extracted from the fMRI maps shown separately on the polar angle and eccentricity maps.

### 2.7 Evaluation of source localization accuracy

The source estimation accuracy of each method was evaluated using three metrics: peak–target distance, polar angle-based retinotopy, and eccentricity-based retinotopy. The metrics are based on an assumption that the primary visual cortex is responsible for the earliest activation peak seen in the sensor-level evoked responses. This assumption is supported by the results from numerous EEG studies, which mostly agree that the peak between 60 and 100 ms can be attributed to the V1 (Di Russo et al., 2002). However, it is likely that the subsequent visual cortices, especially V2 and V3, contribute to the total signal recorded from the occipital cortex and subsequently shift the location of the peak activation. The MEG inverse problem is severely ill-posed making it difficult to separate the contributions from two or more close-by sources (Hagler & Donald, 2014). For the purposes of this study, the accuracy-reducing effect of the crosstalk is expected to influence all localization methods equally.

The exact peak timings between 60 and 100 ms were first computed for all subjects and stimuli individually from the sensor data. A median value was then calculated for each subject using the peak times of the stimulus responses. Values overlapping with the 60 and 100 ms borders were excluded from the median calculation, as they were likely not the local maximum representing the V1 peak but either a point on a slope or a mix of V1 and subsequent visual cortices. These subject-specific median time points were then used in all source estimations. Overall median peak timing was 83 ms with standard deviation of 9 ms.

Quantitative analysis was performed by measuring the geodesic distance between the dipole with the highest amplitude (peak activation) and the target vertex derived from fMRI data for each stimulus. The fMRI-based target points were the same in each comparison regardless of the subject count or tested method. To aid the interpretation of the distance metric, the average geodesic distance between the target vertices in the middle of V1 (corresponding to sectors 9 and 13 inFig. 1) and the other target vertices on the same hemisphere was measured to be 17.8 mm, with the closest neighbor being 6.3 mm away. The vertex displaying the global peak amplitude was selected as the activation location for unilateral stimuli, while one peak from each hemisphere was selected for stimuli on the vertical meridian. For visualization purposes, the location of the primary visual cortex on the*fsaverage*mesh was derived from the location of the calcarine sulcus and cortical folding patterns (Fischl et al., 2007). In FreeSurfer nomenclature, these anatomy-based labels are known as*exvivo*labels.

Qualitative analysis of the localization accuracy was then performed by visually inspecting the peak locations plotted on the*fsaverage*mesh. Each of the peak activation foci was colored based on either the stimulus angle or eccentricity. The results were compared with the fMRI-based retinotopic maps shown inFigure 3. The analysis mainly focused on assessing the relative spatial layout of the peaks. This inspection was done for both polar angle and eccentricity plots of 1, 10, and 20 subjects.

## 3 Results

### 3.1 Comparison of source estimates

Examples of source estimates computed using eLORETA and MWE are shown inFigure 4. While eLORETA and MWE produce individualized source estimates for each subject, averaging the source estimates condenses them into a single estimate representing the whole group. Regardless of the averaging, the minimum Wasserstein estimates are much more sparse and focal compared with eLORETA. The sparsity of MWE is a consequence of the$\ell^{0.5}$norm and it is enforced by the parameter optimization method, which aims for a set number of active source points. The eLORETA-based estimates on other hand are very spread with all subject counts. The estimates also differ by their peak amplitudes. Estimates computed using eLORETA have similar magnitudes around$1\cdot 10^{-11}$Am while the peak MWE amplitudes for the same data vary between1and$1\cdot 10^{-8}$Am.

**Fig. 4.**
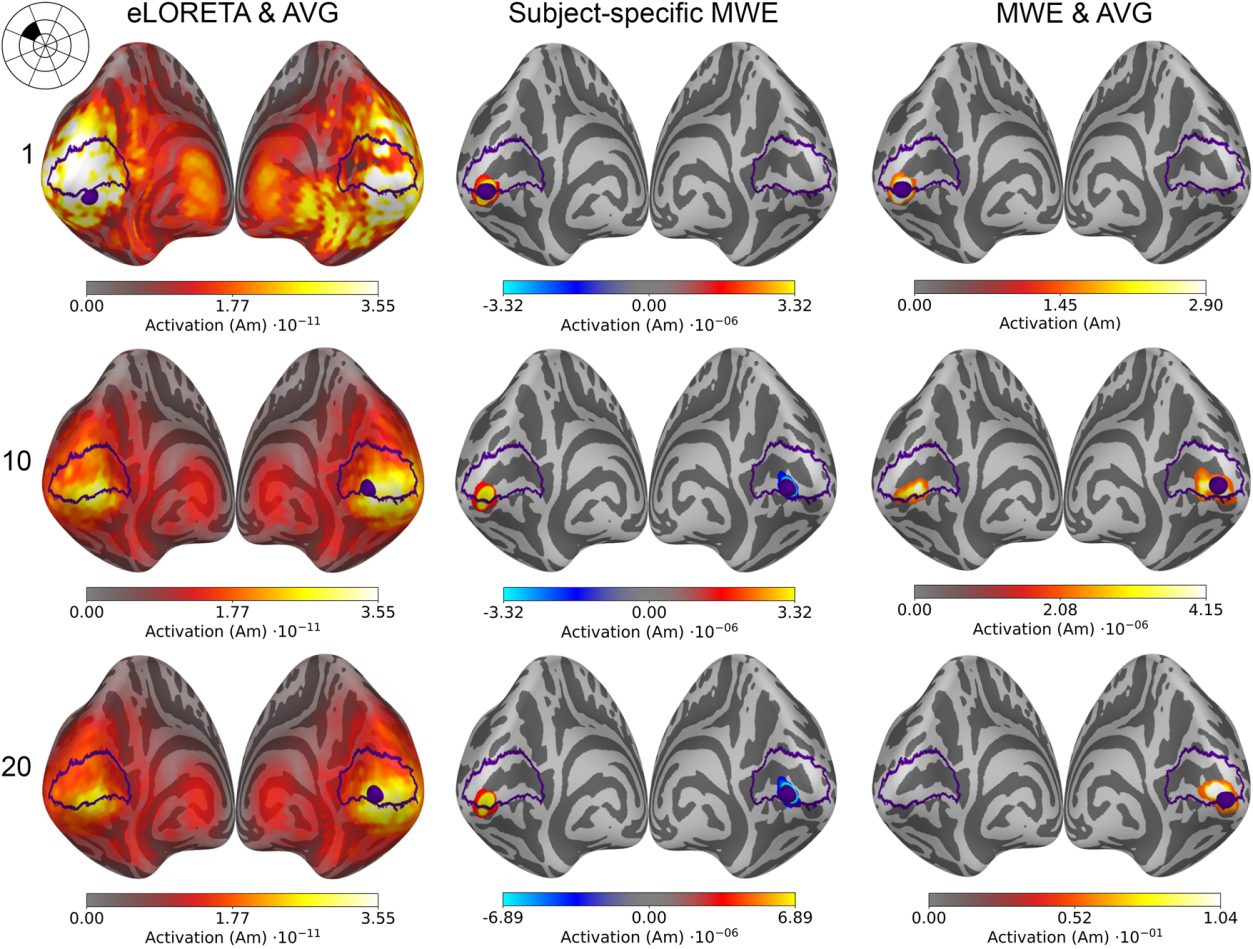
Source estimates of a visual stimulus in the middle of the upper left visual quadrant (sector 16, shown in the top left corner of the figure) for 1, 10, and 20 subjects. All estimates are plotted on an inflated*fsaverage*mesh with no thresholding. The V1 outlines and the peak activation locations are marked with purple.

Based on literature and earlier retinotopic mapping studies, the unilateral stimulus in the middle of the upper left visual quadrant (sector 16 inFig. 1) is expected to activate the lower portion of the calcarine sulcus on the right hemisphere. Similar activity levels are estimated in both hemispheres of eLORETA-based source estimates, but the peak activations are located approximately near the correct locations.

The minimum Wasserstein estimates display bilateral activation with more than one subject, but the amplitudes on the right hemisphere are considerably higher. As with eLORETA, the peak activation is located on the wrong hemisphere for a single subject, but increasing the subject count moves the peak near the correct location on the right hemisphere. Averaging the sparse subject-specific estimates blurs and spreads the active patch a little as the results from individual subjects do not overlap perfectly. Despite this, the estimated patch of activity is near the expected location, and the accuracy is improved by increasing the subject count. The estimated source amplitudes vary between the subjects, causing the difference between the subject-specific and averaged MWE amplitudes inFigure 4.

Source estimates for all 24 stimulus areas are shown inFigure 5for averaged eLORETA,Figure 6for subject-specific MWE, and inFigure 7for averaged MWE. These figures illustrate the progression of the activation hot spot in response to the location of the stimulus. This is especially evident with minimum Wasserstein estimates, as the sparse estimates are mostly contained on the expected hemisphere. The signal is also weaker for the stimuli in the outer eccentricity circles and the upper visual field, as the sources are farther away from the sensors. The effect is best captured in the averaged eLORETA plots. Additionally, the averaged MWE plots inFigure 7illustrate the considerable differences between the individual source estimates. The active patches in the source estimates are spread over a much larger area compared with the estimates of individuals.

**Fig. 5.**
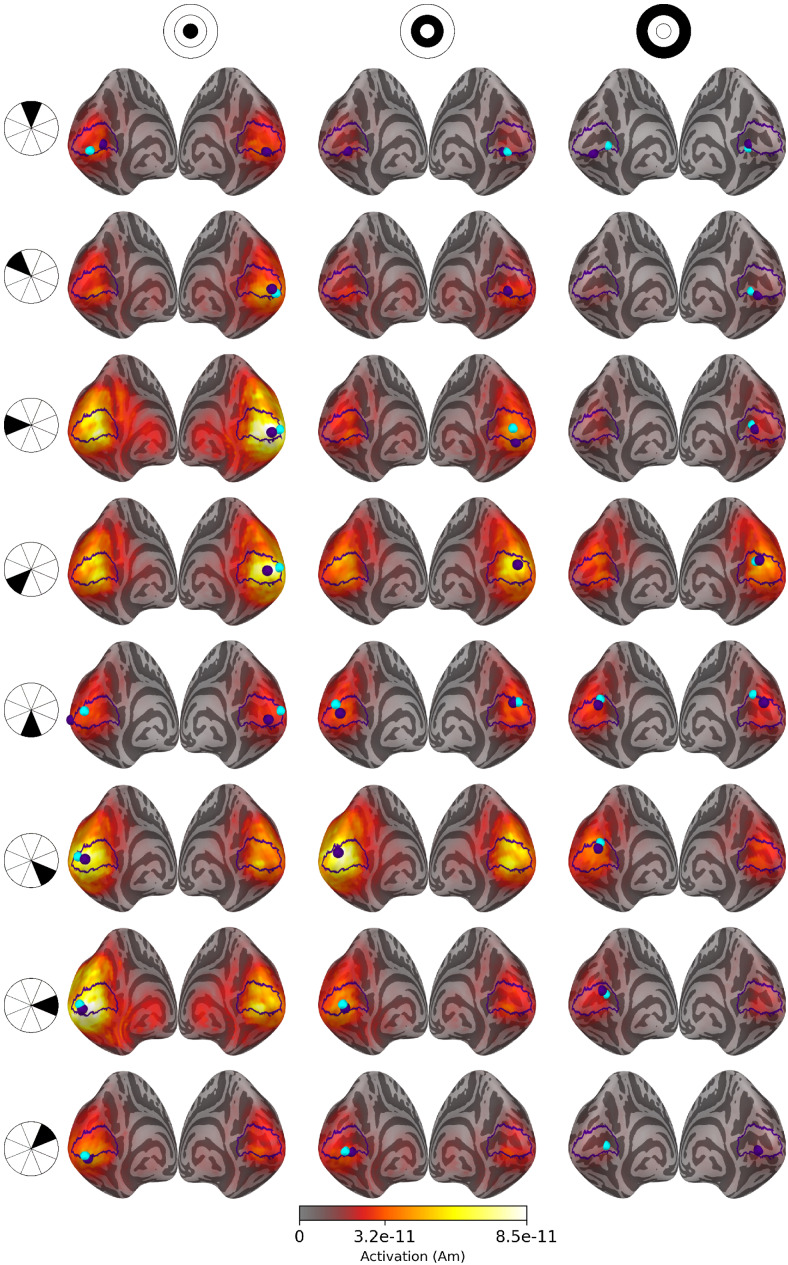
Source estimates for averaged eLORETA with 20 subjects. Columns correspond to eccentricity rings and the rows correspond to the polar angle wedges. Peak values and V1 labels are highlighted in purple and the fMRI target points in cyan. The variation in signal strength is evident from the lower amplitudes in the estimates for outer eccentricity rings and upper visual field stimuli.

**Fig. 6.**
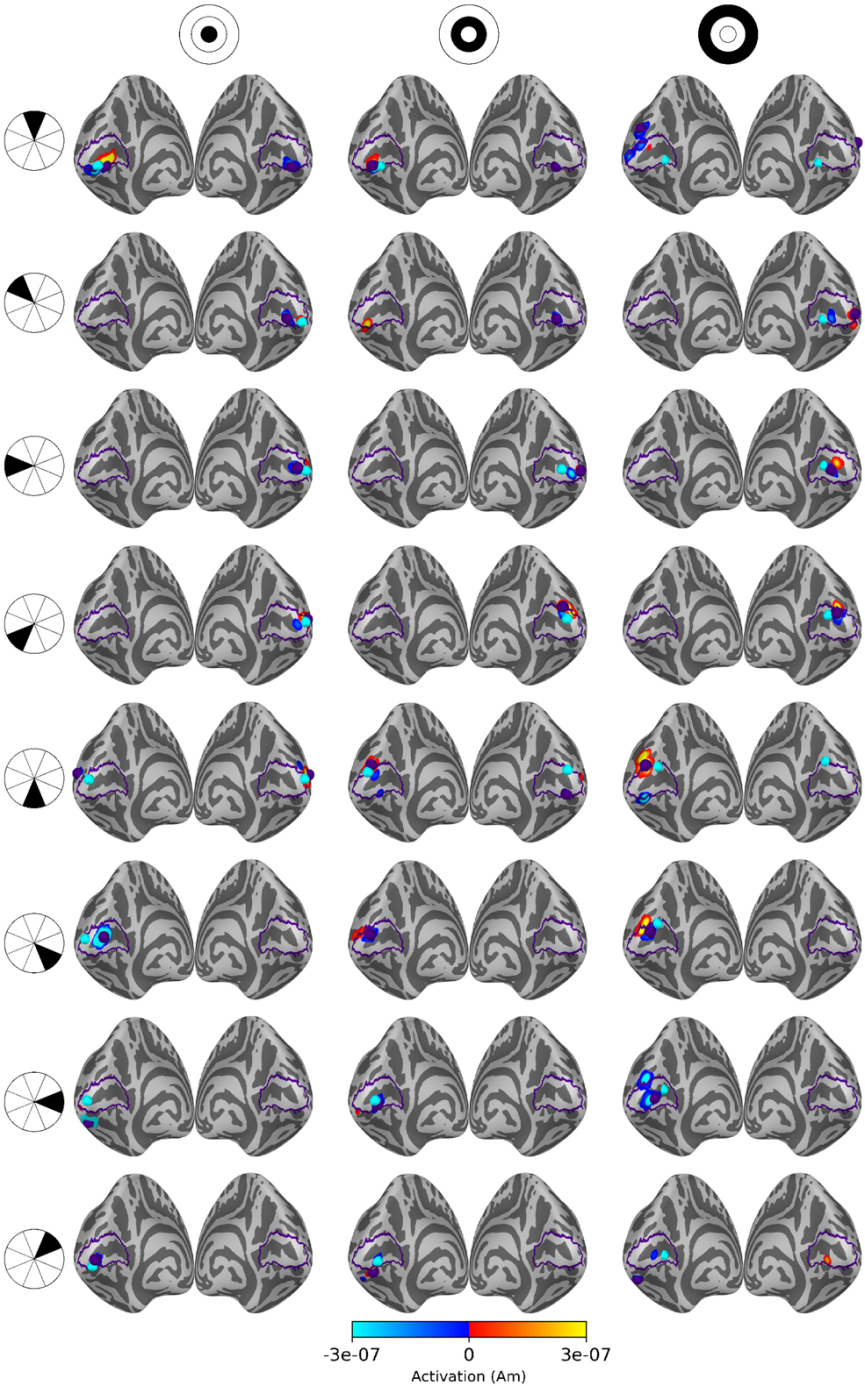
Source estimates for subject-specific MWE computed with 20 subjects. Columns correspond to eccentricity rings and the rows correspond to the polar angle wedges. Peak values and V1 labels are highlighted in purple and the fMRI target points in cyan. The amplitudes exceed the color bar limits in many of the estimates. A low threshold value was selected to show the full extent of all estimates, as the values differ by a factor of$10^{8}$.

**Fig. 7.**
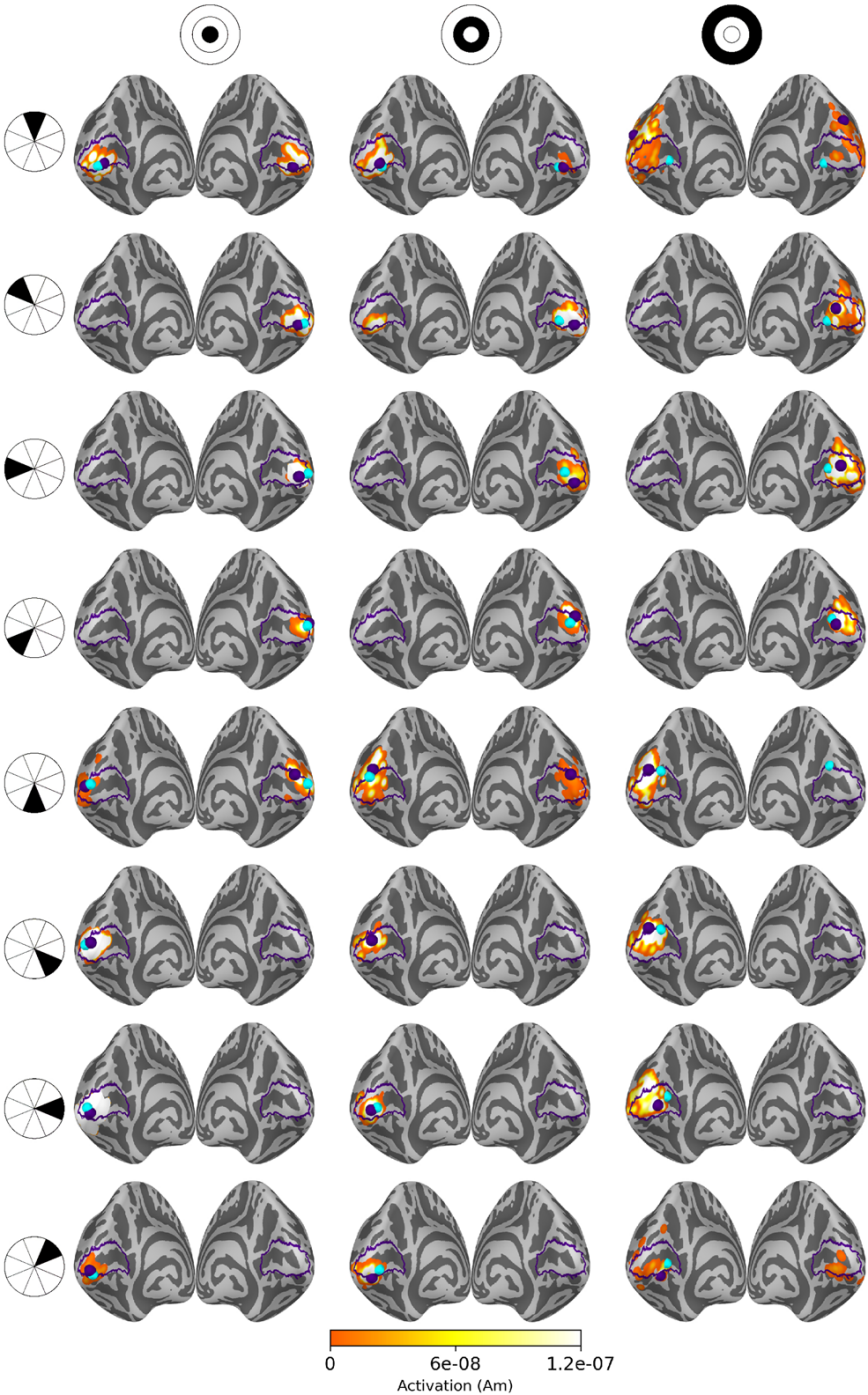
Source estimates for averaged MWE computed with 20 subjects. Columns correspond to eccentricity rings and the rows correspond to the polar angle wedges. Peak values and V1 labels are highlighted in purple and the fMRI target points in cyan. A low threshold value was selected to show the full extent of all estimates, as the values differ by a factor of$10^{8}$. The source estimates are more spread compared with the subject-specific estimates, demonstrating the intersubject variability in the results.

### 3.2 Geodesic distances between peak activations and V1

The localization accuracy was analyzed by measuring the geodesic distance between the target vertices derived from the fMRI retinotopic maps and the peak activation locations on the*fsaverage*mesh. The results of these measurements are presented inTable 1andFigure 8. Peaks were considered outliers if the distance between the peak and the V1 label was over 80 mm or the peak was located on the wrong hemisphere. The outliers were replaced with a value of 80 mm (Winsorized) in the mean, median, and standard deviation calculations. The number of outliers varied between 1 and 2 with eLORETA and between 1 and 5 with MWE.

**Table 1.** Winsorized mean, median, and standard deviation statistics of the geodesic distances between the peak activations and the fMRI-based target vertices for the compared methods.

	eLORETA and AVG			Subject-specific MWE			MWE and AVG		
N	Mean	Mdn	Std	Mean	Mdn	Std	Mean	Mdn	Std
1	24.7	20.0	21.9	28.7	20.3	25.5	28.7	20.3	25.5
5	15.4	11.4	15.2	21.3	11.6	24.0	19.4	10.6	23.1
10	14.9	13.1	14.2	18.4	13.7	18.5	16.3	10.9	18.0
15	13.7	6.6	19.3	24.9	14.6	25.5	23.2	12.4	25.5
20	12.1	8.3	14.4	19.7	13.5	19.3	15.1	8.2	19.2

All numbers are presented in millimeters.

**Fig. 8.**
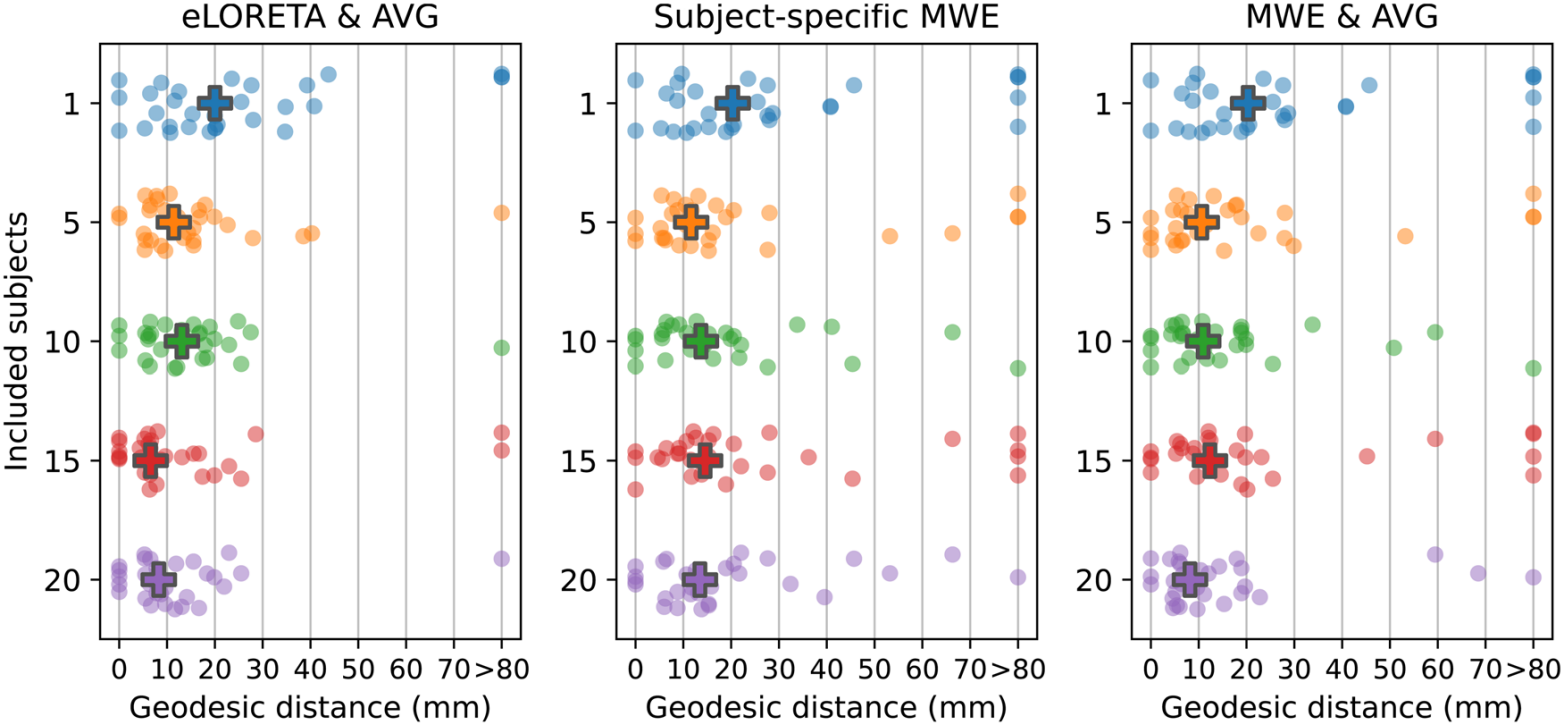
Geodesic distances between the peak activations and the fMRI-based target vertices. Each dot represents 1 of the 24 areas in the visual stimulus. The group medians are drawn with a cross. Peaks localizing over 80 mm away from the V1 or on the wrong hemisphere are considered outliers and their distance values have been replaced with 80 mm (Winsorized) in the median distance calculations. Left: Distances for eLORETA with source-space averaging. Center: Subject-specific results for minimum Wasserstein estimates (Subject 9). Right: Results for source-space averaged minimum Wasserstein estimates.

The mean, median, and standard deviation statistics decrease for all methods when the number of subjects is increased from 1 to 10. The most prominent changes are observed between one and five subjects after which the metrics fluctuate as new data are added to the set. Compared with subject-specific results, the five-subject median distances are reduced by 8.6 mm (43%) for averaged eLORETA, 8.7 mm (42.9%) for subject-specific MWE, and 9.7 mm (47.8%) for averaged MWE. With 10 subjects, the median distances are reduced by 6.9 mm (34.5%) for averaged eLORETA, 6.6 mm (32.5%) for subject-specific MWE, and 9.4 mm (46.3%) for averaged MWE when compared with individual solutions. Considering that on average the targets in the middle of V1 (sectors 9 and 13) are 17.8 mm away from their neighbors, many of the peaks are likely localized to wrong retinotopic areas especially with low subject counts. After 10 subjects the median distances stay within 25% of the 10-subject values for MWE-based estimates, while averaged eLORETA results see a decrease of 49.6% between 10 and 15 subjects.

Increasing the subject count moves the averaged eLORETA peaks closer to each other as seen in the standard deviations of the peak–target distances. The standard deviations are reduced by 6.7 mm (30.6%) for eLORETA, by 1.5 mm (5.9%) for subject-specific MWE, and by 2.4 mm (9.4%) for averaged MWE when the subject count is increased from 1 to 5. With the exception of the 15-subject results, the averaged eLORETA standard deviations are further reduced by up to 1 mm (6.6%) as the subject count is increased toward 20. The standard deviation for subject-specific MWE is reduced by up to 5.5 mm (22.9%) and for averaged MWE by up to 5.1 mm (22.1%)

### 3.3 Peak activation locations

The retinotopic organization of peak activation locations was examined by plotting the peak locations on an inflated cortex and coloring them based on their polar angle and eccentricity. For a visual reference, an anatomy-based label of the primary visual cortex is drawn with a purple outline. A single global peak was selected for unilateral stimuli while the vertical meridian stimuli were given one peak per hemisphere. As the activation peaks for different stimuli can be localized to the same vertex, the size of the bubbles is adjusted based on the number of overlapping peaks. The improvements shown in these plots support the distance metric results. With increased subject counts, the peaks converge toward V1 and the retinotopic organization of the peaks becomes more evident. For reference, the locations of the peaks can be compared with the fMRI targets shown inFigures 3and5–7.

The peak activation charts are plotted inFigure 9based on the polar angle of the stimulus and inFigure 10based on their eccentricity. For an individual subject, the eLORETA peaks are scattered around the occipital lobe with an individual outlier visible on the medial surface of the right parietal lobe. Already with 10 subjects the majority of the peaks are inside or very close to the primary visual cortex label. A level of retinotopic organization can be seen especially in the eccentricity plot. The foveal stimulus regions, shown in blue, are located close to the occipital pole. The more peripheral stimulus regions, shown in yellow and red, are represented farther away from the occipital pole on the medial surfaces of the hemispheres. The results are further improved with 20 subjects and the peaks follow established retinotopic maps for the most part, especially on the left hemisphere.

**Fig. 9.**
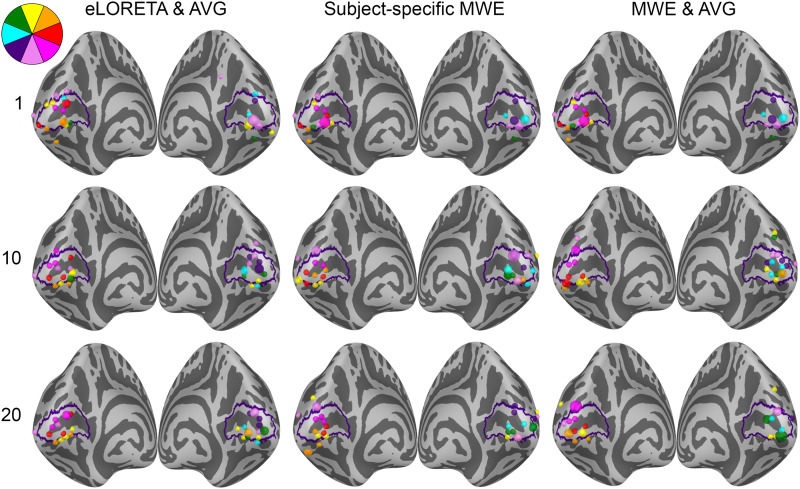
Polar angle-based peak activation location charts for each analysis method with 1, 10, and 20 subjects. Each dot corresponds to a stimulus area except for the stimuli on the vertical meridian, which have two points per area. The size of a dot signifies the number of peaks localizing to the same vertex. The peaks appear more organized with increasing subject counts.

**Fig. 10.**
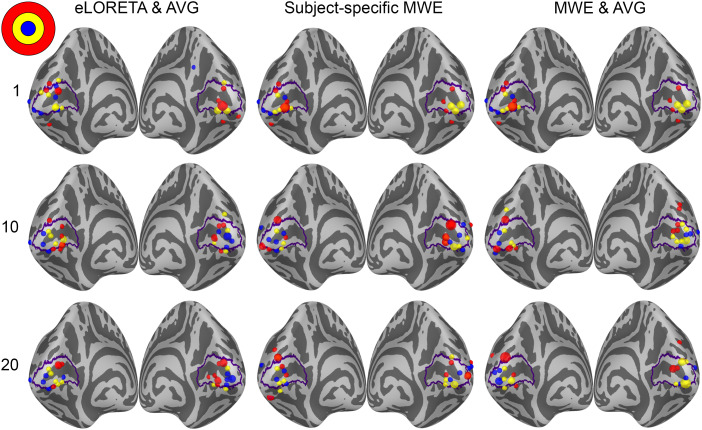
Eccentricity-based peak activation location charts for each analysis method with 1, 10, and 20 subjects. Each dot corresponds to a stimulus area except for the stimuli on the vertical meridian, which have two points per area. The size of a dot signifies the number of peaks localizing to the same vertex.

When the minimum Wasserstein estimate is computed for an individual subject, the peaks are slightly less dispersed compared with eLORETA. Increasing the subject count from 1 to 10 brings the peaks closer to the V1 label especially on the right hemisphere, but a number of them are still located outside its borders. Increasing the subject count further to 20 offers no apparent improvement distance wise compared with the 10-subject chart nor is there an observable improvement in the retinotopic organization of the peaks. The results are not as good as with eLORETA, but they are nevertheless improved by pooling the data from more subjects.

The source-space averaged MWE offers progression similar to eLORETA and subject-specific MWE. As with averaged eLORETA, including 20 subjects provides the best results, although MWE’s accuracy does not meet the same level despite the source-space averaging. The 10- and 20-subject results are very similar between the subject-specific and averaged minimum Wasserstein estimates. Averaging the MWE results provides a minor improvement with 20 subjects, which is most prominent in the eccentricity charts.

## 4 Discussion

The objective of this study was to evaluate the performance of a selection of multisubject analysis methods in a retinotopic mapping task. The three tested methods included eLORETA with source-space averaging, minimum Wasserstein estimates, and MWE with source-space averaging. In contrast to the existing literature on multisubject inversion, we focused on real measurement data and a task, which has proven challenging for MEG. By increasing the number of subjects in our analysis pipeline, the spatial accuracy was improved for all methods. Moving from 1 to 10 subjects, for example, reduced the median distances between the peak activations and fMRI-based targets by 33–46%. The most significant reductions in the median distances were observed between one and five subjects, after which the distance metrics effectively level off. However, increasing the subject count still further moved the peaks closer to each other and their expected retinotopic locations within V1.

Analyzing the peak locations visually also supported the distance metric results. Increasing the subject count allowed for more accurate mapping of the different stimuli as evidenced by the increasingly organized locations of the peak activations within V1. The progression over the subject counts was the most pronounced with averaged eLORETA, as there was a clear difference between the 1-, 10-, and 20-subject results. Especially the 20-subject chart followed the expected retinotopic maps of V1 quite well. Improvements were also seen with subject-specific and averaged MWE when the subject count was increased, but the accuracy did not reach the level of averaged eLORETA. Additionally, the difference between the 10- and 20-subject results was not as high with MWE-based estimates compared with eLORETA.

The results are in line with previous studies on multisubject analysis of MEG data. For example,Larson et al. (2014)tested both simulated and real-world scenarios using sLORETA and source-space averaging. In their simulations, the localization accuracy improved by around 35–40% by increasing the subject count from 1 to 20. In a real-world scenario with auditory N100 responses, the centroid error was decreased by 7 mm (∼46%) when the subject count was increased from 1 to 10. The methods based on multitask learning also benefit greatly from a higher subject count. For example,Lim et al. (2017)employed subject-specific ROIs derived using fMRI localizer tasks as the source space for group lasso. In their simulations, the area under curve (AUC) increased by 45–100% when 16 subjects were considered instead of 1 subject. Compared with conventional minimum-norm estimates or MWE, the size of the ROIs limits the spatial resolution and the all-or-none nature of the method conceals the individual signatures in the source estimates.Janati, Bazeille, et al. (2020)also obtained similar results from their simulations with MWE. Moving from 2 to 16 subjects reduced the generalized Wasserstein distance by roughly 45% while the AUC increased by about 60%. The previous studies also display the saturation point at roughly 10 subjects, after which the distance metrics start to level off (Janati, Bazeille, et al., 2020;Larson et al., 2014;Lim et al., 2017).

Improvements in the spatial accuracy have been attributed to anatomical differences between subjects and increase in spatial information when multiple measurements are combined (Janati, Bazeille, et al., 2020;Kozunov & Ossadtchi, 2015;Larson et al., 2014). These effects manifest themselves, for example, through point-spread function overlap at the true activation location. Multisubject inversions might benefit from a higher SNR compared with individuals, as they have a higher number of effective samples. The increase in information content has been quantified byKozunov and Ossadtchi (2015), who reported a threefold increase in combined leadfield matrix rank when nine subjects were considered instead of one subject. By now the multisubject analysis seems to benefit from the variability between the subjects, but an important point to note is the variable quality of the MEG recordings. The differences in sensor- or source-level signal-to-noise ratios among the subjects were not significant, but the combined effects of variable data, coregistration, and forward model quality might nonetheless help to explain the decrease in accuracy in our results when comparing 5- and 10-subject source estimates.

The MWE algorithm can be augmented to include the concomitant estimation of subject-specific noise level, which could potentially improve the localization accuracy and amplitude estimation. Here the simplified version of the method was used due to issues with parameter optimization and numerical stability when the concomitant estimation was enabled. The peak amplitudes estimated by the minimum Wasserstein estimates varied by a factor of$10^{8}$, which has a notable effect on the source-space averaging results. While the source-space averaged MWE fared better in the distance metrics compared with the subject-specific MWE, the results were likely driven by the high-amplitude individuals instead of being a faithful representation of the group results. Averaging sparse source estimates may not be as robust a method for forming group results as with widely spread MNE results, since the active areas are less likely to overlap.

Previous publications on retinotopic localization with MEG are limited in numbers, group sizes, and extent of the stimuli. Nevertheless, the spatial accuracy of MEG has shown promise in visual tasks when evaluated against fMRI-defined retinotopic maps (Cicmil et al., 2014). For example,Moradi et al. (2003)achieved a mean localization error of under 5 mm for sources inside the primary visual cortex. Detailed retinotopic maps have been produced using magnetoencephalography by, for example,Nasiotis et al. (2017). Retinotopically constrained source modeling has also been used to estimate the activation patterns of visual cortices (Hagler & Donald, 2014;Inverso et al., 2016). Additionally, combining MEG with EEG has been demonstrated to improve the source localization accuracy with a polar angle-based visual stimulus (Sharon et al., 2007).

Moving toward more automated and accurate multisubject analysis pipelines could be especially beneficial in the context of visual neuroscience, as accurate mapping is often a result of manual work on a small number of subjects. In an optimal situation, combining the data from multiple subjects would improve the results, generalize to different stimulus types, and decrease the amount of operator intervention required. On the other hand, one should be aware that the multisubject analysis methods are not applicable to all experimental designs. For example, averaging the source estimates prevents statistical testing against the mean, as the individual source estimates are lost in the process. The methods might also introduce a bias or produce incorrect activation patterns if the true sources are inconsistent between the subjects. Likewise the inconsistencies and errors in tissue segmentation and forward modeling between the subjects can have an effect on quality of the results. For the purposes of this study, we have considered the contributions of these errors to be similar between the source estimation methods. Joint analysis methods producing individualized results, such as the minimum Wasserstein estimates, could in theory produce better results compared with simple averaging as they are designed to have room for these intersubject inconsistencies instead of forcing them to a common activation pattern.

The results are influenced by the choice of metrics, which is highlighted by our peak activation location plots. Despite the median distances remaining similar between 10- and 20-subject estimates, the plots inFigures 9and10show noticeable improvement in the organization of the peaks. In this study, we examined the V1 peak activation by selecting the time point with the highest amplitude between 60 and 100 ms. However, the accuracy-reducing effects of V2 and V3 activation cannot be entirely ruled out by temporal constraints, as there is a considerable level of activity on V2 and V3 at the time of V1 peaking (Hagler & Donald, 2014). The extent of the stimulus responses is also limited to individual points, ignoring the specificity of the estimates and any coincident activation centers apart from the bilateral responses to vertical meridian stimuli. Examining, for example, the top 5% of active vertices would likely yield different results especially with the widely spread minimum-norm estimates. eLORETA estimates high levels of bilateral activity with unilateral stimuli, while the activations in minimum Wasserstein estimates are very much focused on the visual cortex. Visual assessment of the source estimates also shows that MWE estimates the overall activation on the correct hemisphere better than averaged eLORETA.

While a number of joint analysis methods have been published, it is a relatively new concept in MEG context. Majority of the comparisons are focused on simulations and the tasks and metrics are not consistent between the publications. Additional systematic evaluations and comparisons with real data would be beneficial, as would be the inclusion of the temporal dimension. After all, temporal resolution is one of the key strengths of electrophysiological methods.Kozunov and Ossadtchi (2015)andLim et al. (2017)address the source time courses, but otherwise the focus has been on individual time points and simulated localization accuracy. In terms of spatial accuracy, our results support the hypothesis that the increased information content and variation in data provided by multisubject datasets can be leveraged for improved results.

## Data Availability

The analysis pipelines for preprocessed and averaged stimulus responses are publicly available athttps://github.com/PaavoHietala/IN-multi-subject-2024/. The MEG and MRI data used in this study cannot be fully anonymized, hence sharing the data is prohibited by the Finnish data protection legislation.
